# The Wisdom Teeth

**Published:** 1886-04

**Authors:** J. D. Thomas


ARTICLE III.

### THE WISDOM TEETH.

BY J. D. THOMAS, D. D. S.

[Read before the Odontological Society of Pennsylvania, January 2d, 1886.]

The troubles arising from the growth and retarded eruption of the wisdom tooth, which appears to be governed by laws entirely its own, are so many and so severe in their character, that it seems strange so little has been done by the profession at large in mitigation of the evil, other than the heroic treatment,—extraction. From malposition and crowding, it is the source of untold distress, over which the patient has no control, and is more perplexing to the dentist than any other tooth presented for his skill. It shows itself sometimes with its crown pointing directly towards the tongue, with roots lying almost diagonally across the jaw, and sometimes so far embedded in the cheek that its lingual surface will be on a line with the buccal surface of the anterior molars; at another time it will be in what may be considered its normal position, but if an inferior one, situated so far back in the angle of the jaw that perfect eruption is impossible. The most common and trying position for both patient and operator, however, is when it comes obliquely from the angle of the jaw, with its crown pressing firmly against the posterior surface of the second molar. These are the cases which cause so much suffering, not only from the growth of the tooth itself, but frequently from injury to the second molar, while its extraction generally entails still further suffering for days, and perhaps weeks, after the operation, with a possi bility of ultimate loss of the second molar too.

There are three forms of suffering produced by the wisdom tooth, the cause for two of which may be defined by one word, "pressure." In many instances from defective structure, the tooth will become decayed to the pulp almost as soon as it makes its appearancc in the mouth; and this form of difficulty will present itself as an ordinary case of tooth-ache, with perhaps neuralgic accompaniments, which, according to indications, may be treated by remedial application, or extraction advised. The latter course will be referred to later on.

Of the two forms, the cause of which is attributed to pressure, the principal one is abscess in the angle of the jaw, sometimes of slight degree, but more frequently of such serious nature as to involve considerable swelling of the cheek and maxillary glands, and perhaps extending into the soft tissues of the throat, so as to cause entire cessation of mastication, greatly impeding the swallowing of even liquid articles of nutriment. The results attending these conditions are sometimes most serious. Besides the necessary confinement of the patient, should the abscess break on the outside of the cheek, there is the scar which he has always to carry. The prostration produced by suffering and inability to take proper nourishment will take a long time to recover from completely; and I have seen cases in which not only the preceding molar or molars have been sacrificed, but a considerable portion of the bone has become necrosed also, and in a few instances the effect upon the masseter muscle has been such that perfect motion to the lower jaw has never been regained. Such are some of the varying phases of condition which this tooth causes to exist. If our patients would come for assistance at the first approach of the difficulty a good deal of their suffering might be averted, but as a rule they come as a last resort, after the application of all the home remedies they know of, and perhaps after the efforts of their family physician has failed to afford relief.

The third form, as caused by pressure, presents itself in a condition of extreme suffering of apparent neuralgia. The patient will complain that he has been the victim of slight attacks for sometime, but not of sufficient severity to cause him much inconvenience; but now the pain has become unbearable, and he thinks he can locate its origin in the partially erupted wisdom tooth. These cases have been found most perplexing, for the reason that careful examination has failed to disclose the slightest defect about the tooth that could confirm the patient's idea that it was the source of his pain. There will not appear to be the least inflammation about the parts, and everything will seem perfectly healthy. The second molar will present a like good condition, and yet the patient is suffering the greatest agony. In one case of this character, upon examination, the inverted wisdom-tooth crown was found pressing directly against the posterior root of the second molar; the only portion visible of the wisdom-tooth, was what in its proper position would have been its posterior surface, and only enough to show how the tooth lay. There was no decay exhibited in either it or the second molar, neither was there the slightest tenderness to tap, or the application of warm or cold. The pain seemed to originate in the wisdom tooth, as near as the patient could describe, though it included the ear and the whole side of the face, yet nothing could be made to appear that would confirm it. Being averse to counsel extraction without a clear understanding that good would result from the operation, the patient was about being advised to look elsewhere for the cause, or at least defer it until later, to see if something might not show itself, to indicate more clearly that he was right, when, upon final examination, it was found that pressure against the second molar, toward the wisdom-tooth, would start the pain. This was convincing. After extracting the second molar there was found, three-sixteenths of an inch from the apex of the root, an opening into the pulp canal, caused by the anterior cusp of the wisdom-tooth, in its lateral position, pressing against the dentine, until by absorption it had penetrated into and was pressing against the pulp, which was much inflamed. Since meeting this condition, I have seen several of like character, sufficient to show that this form of disease is by no means uncommon among the ills to be charged to this unruly organ.

I do not wish to be considered as asserting that these teeth, in their malposition, are always the cause of trouble to the patient. On the contrary, many of them will become fully developed in the jaw and never perfectly erupted, yet will not have caused the slightest inconvenience, though in a paper read before the New York Odontological Society, by Dr. La Roche, and published in the July number of the *Dental Advertiser* a case is described of a gentleman whose mental condition bordered upon insanity, diagnosed to be caused by the retarded eruption of these teeth, and the result of a cure following their extraction, proved the diagnosis correct. From his description, Dr. La Roche seemed to form his opinion entirely from the one fact that the teeth were present in an unerupted state. I have seen a number of cases where patients have suffered in like manner, if not so severe, and the cause has been attributed by themselves and their family physicians to these partly developed teeth; but upon examination there could be nothing found to satisfy me positively that they were the cause of the trouble, and relief was not obtained by their extraction. From this experience, I hold to the opinion that because a wisdom-tooth may be, as we call it, inverted, it is not always the cause of the suffering from both the aural and facial neuralgia attributed to it, any more than an unerupted tooth anywhere else in the mouth. We all know that it is not an uncommon occurrence to see among our patients a mouth in which a bicuspid, a lateral, or even an eye tooth is wanting, even in persons advanced in years, and upon extracting an adjoining tooth the missing one is found fully grown, but so crowded in the jaws that eruption has been impossible, though in its healthy state we would not look for the source of neuralgia in such a tooth. In one case of this kind that came to my care for extraction, the patient was a great sufferer from neuralgia, which was attributed by her physician to her teeth, all of which in the upper jaw were more or less badly decayed. In extracting it was noticed that the canines were absent, but the patient could not recall ever having had them extracted. Upon examination after the operation, the crowns of the eye teeth were distinctly felt, imbedded in the jaw in an almost lateral position. The patient was nearly sixty years of age. Owing to the depth of the position of the teeth, extraction was not advised at that time, but relief was obtained for the space of two months, at which time the neuralgia recurred more severely than ever, and it was decided to extract the eye teeth. There was nothing in the appearance of the gum to lead to the supposition that they were the cause other than the knowledge of their presence in the jaw; but there was hope that their extraction might exercise some influence to give her relief. The cessation from pain lasted only about three weeks after the operation, showing that the teeth were not the cause of the neuralgia.

To return to the consideration of the wisdom teeth. The prescribed treatment in most cases is extraction, and there is no doubt that many people would be better off without them; but extraction, besides being difficult for the operator, becomes a matter in so many instances of such serious consequence to the patient that there ought to be such consideration given to the subject that would result in sparing him much that he now has to bear. I do not mean that extraction, in all cases, is productive of so much annoyance. There is, as a rule, little to apprehend with the superiors, and many of the inferiors are accompanied with no more after pain than any other tooth, but I refer particularly to the irregular ones of the lower jaw.

In the first place they grow out of the solid bone, right at the angle, where there is no alveolar process. The bone broadens here and is very dense, so we have solid bone on both the buccal and lingual sides, the ramus over the roots and the second molar in front. In the second place, they invariably grow with curved and distorted roots, so, to extract them any how you will, it can be done only by great effort and with subsequent suffering on the part of the patient. If it should be a badly decayed one, the chances are that there will not be strength enough in the tooth to bear the force necessary to loosen it, in which case it becomes necessary to cut it down as deeply as possible, extract the pulp and leave the remaining portion to work up to the surface for future operation; or get over the bone and cut through it, with the alternative of extracting the second molar to give a better opportunity to extract the wisdom-tooth. In either case the patient will have a very sore mouth, which will require treating for perhaps several days. If the case be one causing trouble from pressure alone, extraction is likely to be attended with better success, but the immense strain upon the parts already inflamed by the process of eruption, will cause a still greater amount of inflammation, with perhaps a severe abscess as a result. It is always necessary to have the patient pay repeated visits, or else to visit them at their homes for after treatment. In a few cases where they have negleeted to pay attention to instructions and have applied home remedies in the shape of poultices to the outside of the face, or have called in their family physician, I have been invited to pay for his services, and even threatened with suit for damages for loss of time and suffering. Being a witness to so much that is unsatisfactory, I have been led to advise in many cases the removal of the second molar in preference to disturbing the wisdom-tooth. If the latter is growing straight, it will move forward and prove a very useful organ, and should it be one with the crown pressing against the second molar, I prefer the anterior tooth, for the reason that from the position of the wisdom-tooth its removal will cause much friction against the posterior root of the second molar. I have seen several in which the pulp has died, accompanied by ultimate loss of the tooth as a result. To relieve the pressure is the object sought, and in a case where the pain of extracting the wisdom-tooth is likely to be severe, the removal of the second molar has been found to give the greater satisfaction.

After extraction is decided upon, how it shall be done is a question for each operator to elect for himself. I lay no claim to superiority, for my own methods over those of others, preferring the general success attending the operation should speak for itself. I read an article in the February *Dental Cosmos* in which the writer claims at this day, that the key will extract certain teeth "more readily, easily, quickly and successfully than any other instrument ever invented." I am willing to admit that it makes very little difference with what instrument it is done, if only the operator has become accustomed to its use, and it suits his purposes best. A former professor in one of our dental colleges has said that to be a proficient dentist, one should be able to fill a tooth with a rusty nail; upon the same teaching a man ought to be able to extract teeth with a pair of gas plumber's pliers; but I cannot see the policy of becoming so accustomed to their use that they will be claimed as the best instrument extant for that purpose. Some operators recommend the use of the Physic forceps for the extraction of these teeth, but I fail to see in them any advantage over the regular forceps, but probably, like the key, it may be from ignorance of their proper use. It is necessary that instruments should be so constructed in their beaks as to insure a firm hold upon the tooth without slipping; there should be as little curving in the handles as possible, so as to bring the force in extracting as near a direct line from the hand to the tooth as possible, and the operator should assume a position with the patient that will insure the greatest amount of force with the least physical effort. To the wisdom-tooth the exertion must be applied to loosen it in its socket before any attempt at pulling should be made, after which its removal depends upon whichever way the position offers the best advantage. In cases where it is locked in by the second molar after loosening, it can be turned and worked in its socket until the bone on either side is sufficiently distended to allow the tooth to be taken from under the second molar; but it is just this distention of the bone that comes after trouble. Sometimes, instead of giving, a considerable portion of the bone will break away on the sides to which the force expended. This will cause no permanent injury, provided the broken portion is removed and the wound left to heal without irritation; but it adds to the serious consequences of extraction as a remedy for these conditions.

I would suggest judicious extraction for children from ten to fourteen years of age, as a prevention of the evils produced by the retarded eruption of these teeth. Though not an advocate of the removal of all sixth year molars, I am confident there are thousands whose mouths would be benefitted by the practice.

Never until a month ago have I seen a case of difficult eruption of the wisdom-tooth, where there had been a tooth extracted on its side. In that case all four of the second cuspids had been extracted for regulating, and yet the right lower wisdom had not room to grow in place; but that is the only one I have ever noticed, and there is no doubt, if upon careful examination of the mouths of young persons, the removal of a tooth was advised, all around, either the first or second molar, or one of the bicuspids, as good judgment would dictate, that the operation would be productive of great good to the patient; and would entirely obviate the difficulty attending the crowding of the wisdom-teeth, taking, of course, due consideration of the development of the jaw, the size of the teeth generally, and the prospect of future growth.

DISCUSSION.

Dr. Chupein.—I have seen so much benefit result from the extraction of the sixth year molars, and I think in the majority of cases it is the best thing to do, since it gives ample room for the eruption of the wisdom teeth. These decay frequently, not because they are weaker than the other teeth, but because their crowded condition causes the gums to fold over them and form pockets for the retention of food.

Dr. D. Neall.—By means of the Physic forceps I have repeatedly pushed out the wisdom-teeth; years ago I frequently used the key in the extraction of the sixth year molars without soiling the instrument with the blood or other fluids of the mouth.

Dr. Wood.—A gentleman was in my chair this morning with a complete set of good sound teeth. One-third of the lower wisdom-teeth were ground away by the upper ones, apparently the result of an effort upon the part of these lower teeth to assert themselves, and get out of their unnaturally cramped position. I have for years advocated the extraction of sixth year molars, too weak to be anything but an embarrassment in saving of better teeth. I recall one case where I condemned and extracted the lower sixth year molars of a boy about twelve years of age. When I saw him again he was about twenty years of age. I found the second molars largely occupying the position of the absent first molars, and the wisdom teeth fully erupted, and well developed; but there was no indications of the coming of the superior wisdom-teeth.

Dr. Tees.—Troublesome cases of horizontal eruption of the wisdom-teeth are seldom met with in general practice. The specialist meets with them more frequently, on account of the large number of patients demanding extraction. The more general extraction of the sixth year molars at eleven years of age, when presented with approximal decay, would tend to obviate the painful eruption of the wisdom-teeth.

Dr. Darby.—I most heartily endorse all that Dr. Neall has said in favor of the Physic forceps. I have always looked upon them as a most valuable instrument for the extraction of wisdom-teeth. Were it used oftener in a skillful way, less injury would be done to the jaws than by endeavoring to remove such teeth without them.

I would enter my protest against the common practice with some of removing the sixth year molars. I frequently see the bad effects of the too general extraction of these teeth. I am well aware that in some mouths their removal is attended with good results; but to extract them indiscriminately, because they are sixth year molars, and more or less prone to decay, is in my opinion bad practice. I know of one dentist in this city who seems to have doomed all sixth year molars that come under his treatment; and I am frequently seeing the bad results of his practice, often large spaces between the anterior teeth, and in some instances the bicuspids close neighbors of the second molars. We should study a mouth well before deciding to remove from it, the largest and in some respect the most important teeth of the set.—*Dental Office and Laboratory.*

---

ARTICLE IV.

